# Towards a formal framework for integrated design-optimization and control of mechatronicsystems

**DOI:** 10.1177/00368504211037460

**Published:** 2021-11-23

**Authors:** Fariba Rahimi

**Affiliations:** Department of Machine Design, 225274 KTH Royal Institute of Technology, Stockholm, Sweden

**Keywords:** Co-design optimization, IDIOM framework, non-linear dynamics, physical design, system modelling

## Abstract

This paper presents work towards a formal framework to support model-based integrated design and optimization of mechatronic products for early-phase conceptual design. This paper describes an integrated design framework through the introduction of its software implementation and a specific use case. The contribution is to introduce mathematical formalism to define the concepts, semantics, computation rules and system architectures of the formal framework. The advantage of the formal definitions is to clearly expose functionality and the limitations of the design framework and facilitate the software implementation. The modelling capability of the framework is enhanced to include non-linear mechatronic components, such as a two degrees-of-freedom arm. Further, an optimal proportional–integral–derivative control component is added to the software library supporting the framework.

## Introduction

Mechatronic systems are engineered by using a wide range of disciplines and are collaborative in nature. Chen et al.^1^ express that the collaborative development of mechatronic systems is error-prone because contemporary design environments do not allow adequate flow of design and manufacturing information across the domains.

The IDIOM framework presented by the authors^2–4^ facilitates design optimization of mechatronic systems in an early design phase to reduce time and cost consuming debugging and re-design in later design phases. Therefore, the presented method in IDIOM integrates engineering disciplines in a rather early design phase. The method uses a static dimensioning approach for the physical design of the component models as well as dynamic models to analyse behaviours of the entire system including maximum component loads. A genetic algorithm (GA) is employed to optimize the system in terms of size, energy and cost with respect to the developed models in the framework. The framework supports the addition of any relevant models for co-design optimization of mechatronic systems.

**Figure 1.**
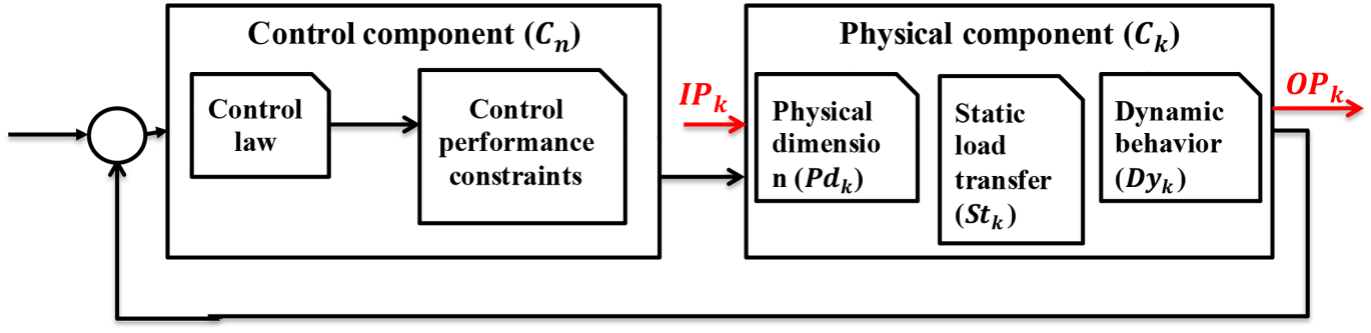
The structure of one physical and one control component in IDIOM.

Figure 1 shows a typical system model in the proposed framework. The framework includes two kinds of component models: physical components and control components. The physical component models describe the classical mechanical components such as actuators, transmissions, and machine elements. Each physical component consists of three main sub-models including physical dimension, static properties and dynamic behaviour models to capture the characteristics of that component for later evaluation. The control component consists of two sub-models for implementing the control law and imposing control constraints on the system.

### An overview of IDIOM framework

The presented IDIOM software toolbox uses an object-oriented programming paradigm.^3^ The component models are defined as class methods that represent objects in the optimization run. When the optimization begins, the software toolbox checks for errors and missing points of the configured system (sanity check). After this, dynamic and static preparation functions of the components are executed to determine the plant model and load the necessary data into the memory (details are presented in the ‘System model computation’ section). In this stage, a parallel pool and workers are employed to achieve the best use of a multi-core computer. The multi-objective function is evaluated and physical dimensions, static properties and dynamic behaviour models are executed. IDIOM can handle both single and multi-objective multidisciplinary design optimization problems and it also features a graphical user interface.^5^ The basic concepts of the method in the IDIOM framework are as follows:

- components, $C_{k}$ and $C_{n}$ , represent physical and control components, respectively,
- requirements as position profiles,
- a composition of K physical components that results in an open chain system composition as $G_{p}=C_{1}⨂C_{2}⨂\cdots ⨂C_{K}$ or in short $G_{p}={⊗}_{k=1}^{K}C_{k}$ ,
- the physical and control components composition results in a closed chain system composition ( $G_{cl}$ ),
- each composition consists of one dynamic input port for a single-input single-output system and multi dynamic input ports for a multiple-input multiple-output (MIMO) system,
- each composition consists of interface connectors for open and closed loop dynamic configuration, and
- each composition includes input ( $IP_{k}$ ) and output port sets ( $OP_{k}$ ).

As shown in Figure 1, the physical component model ( $C_{k}$ ) of component k is a structure of three modelling elements: a physical dimension model ( $Pd_{k}$ ), a static load transfer model ( $St_{k}$ ), and a dynamic behaviour model ( $Dy_{k}$ ), where $C_{k}$ also includes an input port set ( $IP_{k}$ ) and an output port set ( $OP_{k}$ ). A control component ( $C_{n}$ ) includes the control law of the chosen control method and control performance constraints. The necessary definitions are given in the ‘Basics of the supported software framework’, ‘Component level concept’, and ‘Dynamic system modelling’ sections, respectively. The new features which will be presented in the next sections are mainly based on extending the available models in the IDIOM framework, therefore they are implemented by extending the physical dimensioning, dynamic behaviour and static transformation models, used control methods and applied algorithms.

A primary advantage of the original IDIOM framework is to avoid the direct computation of the timed sequence of system responses. The input signals are decomposed into harmonics by Fourier transform and the response of each harmonic is easily obtained from the system transfer function. With the extension presented in this paper, the IDIOM framework contains MIMO and non-linear components and hence the frequency decomposition method is not applicable. The sequence of system response is instead computed directly by the system state-update equation and the input sequences. In the application example of this paper, an optimal proportional–integral–derivative (PID) control method and user-defined control constraints are evaluated using simulation within the optimization evaluation. This has increased the computation time considerably in comparison with the previously presented Fourier-based system behaviour computation. However, the computational time is still on a reasonable level that allows the new methodology to be a valuable tool in the early-phase co-design optimization of mechatronic systems.

The outline of the paper is as follows, Section ‘Introduction’ is an introduction of related work and the contribution. Section ‘Review of related work’ is a literature review of previous studies. Section ‘Basics of the supported software framework’ presents the basics of the supported software framework together with detailed mathematical definitions of each concept and a conceptual case study is also presented to facilitate the comprehension of the definitions by *Illustrative* examples. In the ‘Component level concept’ section, the component level concept is presented formally. Section ‘Dynamic system modelling’ introduces the system modelling approach. In the ‘Optimization’ section, the implemented optimization approach is described. The system model computation together with the pseudo-code for the algorithms is investigated in the ‘System model computation’ section. The related detailed expressions of the case study are presented in the ‘Case study’ section. The results and discussions of the co-design optimization of the presented case study are included and compared with another published method and presented in the ‘Results and discussion’ section. The paper is concluded in the ‘Conclusion’ section.

## Review of related work

The use of multidisciplinary design optimization for dynamic system design is reviewed by Allison and Herber.^6^ Li et al.^7^ derived a general model to mathematically define the concurrent design of a mechatronic system. Based on this model, a concurrent engineering approach, design-for-control (DFC), is formally presented. In comparison with other mechatronic design methodologies, DFC highlights obtaining a dynamic model of the mechanical structure by a structural design and a careful selection of mechanical parameters. Once the simple dynamic model is available, regardless of the complexity of the mechanical structure, the controller design is achieved and better control performance is realized. Nevertheless, their method includes complex models as well as time-consuming iterations.

Gausemeier et al.^8^ presented a graphical method to express the functional principle solution of a mechatronic system called the semi-formal specification language. They applied this method to a vehicle guidance system. A gradient-based framework is proposed by Lee et al.^9^ for optimization of an aerodynamic system and the controller is designed using high-fidelity models. They have considered the system’s general properties such as time scales of the model and used cost function to reduce the computational task of fluid dynamic simulations. They reduced the optimization time by control of the error of gradient which is computed by the optimizer. They provided a few use cases that show their method is applicable for optimizing supersonic vehicle shapes and can handle both sharp and smooth geometry design parameters. However, their method does not cover the detailed physical design of systems.

Delbecq et al.^10^ present a Python framework for the design of embedded mechatronic systems to support the designer in satisfying constraints such as energy consumption, influencing the environment, geometrical integration and reliability. The dynamic simulation through zero-dimensional to one-dimensional models of the system is used to validate the architectural choices. Their optimization approach cannot be applied to the system level even during the early design phases.

Dumlu^11^ proposed a fractional-order adaptive integral sliding mode control scheme to perform a trajectory tracking control of six degrees of freedom (6-DOF) robotic manipulator. Their research is one out of a huge number of other studies that focuses on control of robotic systems where the component design and optimization are neglected.

Chhabra and Emami^12^ used bond graphs and block diagrams to present a concurrent design method. They considered a mechatronic system as an energy system and applied the laws of thermodynamics to specify design criteria. Their paper studies the principles of a multidisciplinary system and the flow of energy and information throughout its different constituents. Subsequently, they introduced a fuzzy logic-based concurrent design framework where they applied it on a 5-DOF industrial robot manipulator. They used a holistic concurrent design approach to convert a multi-objective constrained optimization to a single-objective unconstrained problem.^13^

Domingues et al.^14^ present a design method to achieve size, efficiency, optimal driving performance and thermal characteristics of electric powertrain components. Their methodology finds optimal component combinations based on some requirements. The *driving cycle* behaviour and specific operating modes such as maximum wheel speed are considered. Their method allows the selection of electrical machines, sizing of the gearbox, and the power-electronic converter that are needed to handle the wheel torque and speed requirements. They used their method to optimize an electric powertrain of a passenger vehicle.

An integrated design method called DFC, is proposed by Mohebbi et al.^15^ for a quadrotor unmanned aerial vehicle (UAV) equipped with a stereo visual servoing system. They presented the dynamics and a control model of the quadrotor UAV and its visual servoing system, and later a design process was implemented in four iterations. Subsequently, Mohebbi et al.^16^ proposed a multi-criteria approach for the conceptual design of mechatronic systems. They proposed three different methods using a case study of designing a vision-guided quadrotor drone system. Three different aggregation techniques were used in these methods such as Choquet integral, Sugeno integral and fuzzy-based neural network. They have concluded that even though the Sugeno fuzzy can be a useful aggregation function for decisions under uncertainty, but the approaches using Choquet fuzzy and fuzzy integral-based neural networks are more reliable and precise in achieving results for multi-criteria design problems. Choquet fuzzy integrals are one of the most reliable models which are used in decision theory for multi-criteria decision-making. However, the fuzzy measures have many parameters and are usually complex when only relying on the designer’s intuition. In another study, Mohebbi et al.^17^ compared three methods of fuzzy measure identification tailored for a case study of designing a vision-guided quadrotor drone.

Subsequently, they proposed a fuzzy-based approach^18^ for modelling a unified performance evaluation index in the detailed design phase for a vision-guided quadrotor unmanned aerial vehicle. This performance index is a multidisciplinary objective function that collects all the design requirements from different disciplines and considers the interactions between the objectives and implemented particle swarm optimization algorithm. Their method is a systematic and multi-objective design thinking approach rather than a sequential design method, however, they have used unnecessary complex methods and did not consider integrate physical dimensioning, dynamic behaviour and static properties of the systems in an early phases of design.

Previously mentioned IDIOM framework is a well-developed framework to fill the existing methodology gaps when treating different engineering disciplines in the co-design of mechatronic systems.^19^ IDIOM is capable of dealing with both single and multi-objective multidisciplinary problems. An early-phase co-design method is implemented in IDIOM which can handle multi-DOF linear and non-linear mechatronic systems and yield an optimum solution with respect to defined objective(s) and constraint(s). Physical dimensioning, dynamic behaviour and static properties are considered and models which are simple enough while capturing the main characteristics of the systems are considered. Individual component models are developed throughout the method in IDIOM which allows configuration of system concepts, therefore any system which uses the available developed components is realizable by the method. However, there is yet no formal systematic definition of this framework and its tools. This paper presents formal definitions of the modelling artefacts and methodology of the IDIOM framework. The rigorous and unambiguous descriptions facilitate understanding of the architecture and capabilities of the IDIOM framework and are helpful for software implementation to support the IDIOM framework. The formal definitions, including all modelling artefacts and the computation procedure, facilitate comprehension and software implementation of the framework. Moreover, the modelling capability of the IDIOM framework is enhanced by adding non-linear mechatronic components, for example, a 2-DOFarm. Further, an optimal control component, namely a PID controller is added as a new component model.

## Basics of the supported software framework

**Figure 2.**
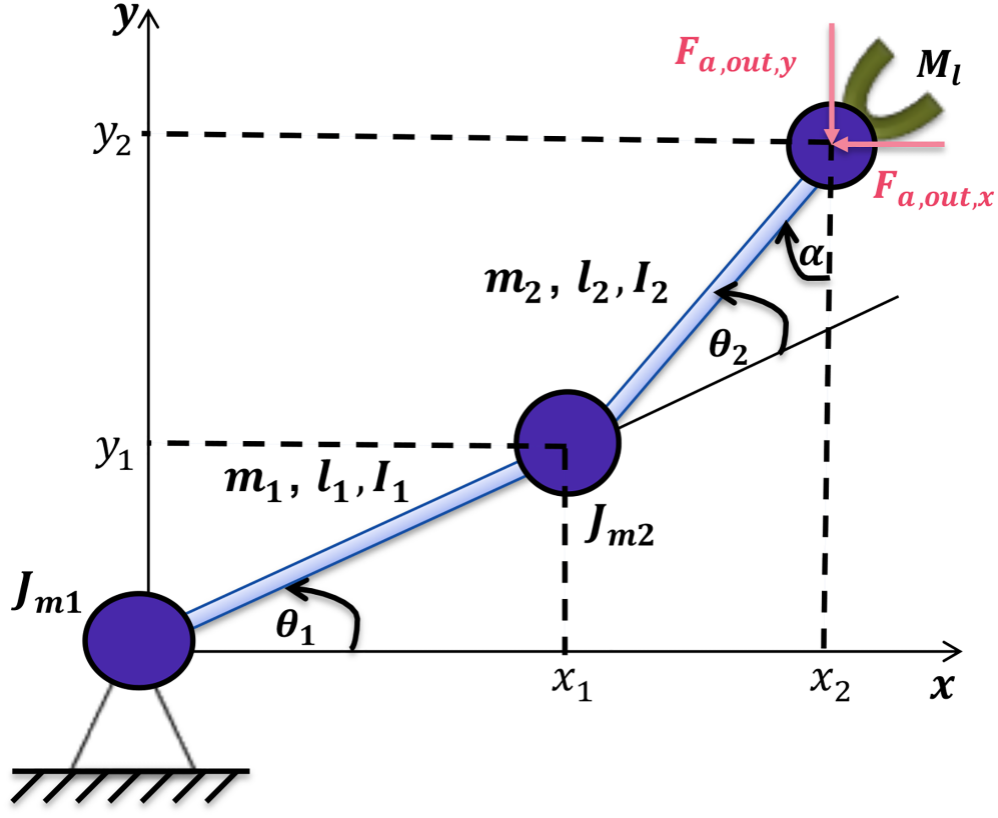
The structure of a two-degrees-of-freedom (2-DOF) robot arm ( y corresponds to the vertical axis).

This section presents formal definitions of all model components of the framework. To assist the reader, the case study in Figure 2 is used to illustrate abstract concepts throughout the paper.

**Figure 3.**
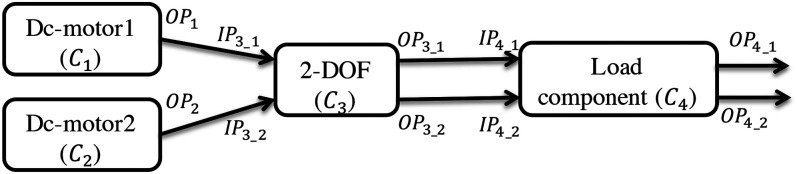
System configuration in IDIOM framework for static analysis.

The case study consists of four physical components ( $C_{1},\ldots ,C_{4}$ ). $C_{1}$ and $C_{2}$ correspond to the first and the second direct current (DC) motors, $C_{3}$ corresponds to the 2-DOF arm as one whole component and $C_{4}$ is the load component as shown in Figure 3.

Let $N_{r}$ be the number of xD-trajectory with respect to the number of actuators in the system, which translates to position profile requirement(s) defined in the output of the system.

**Figure 4.**
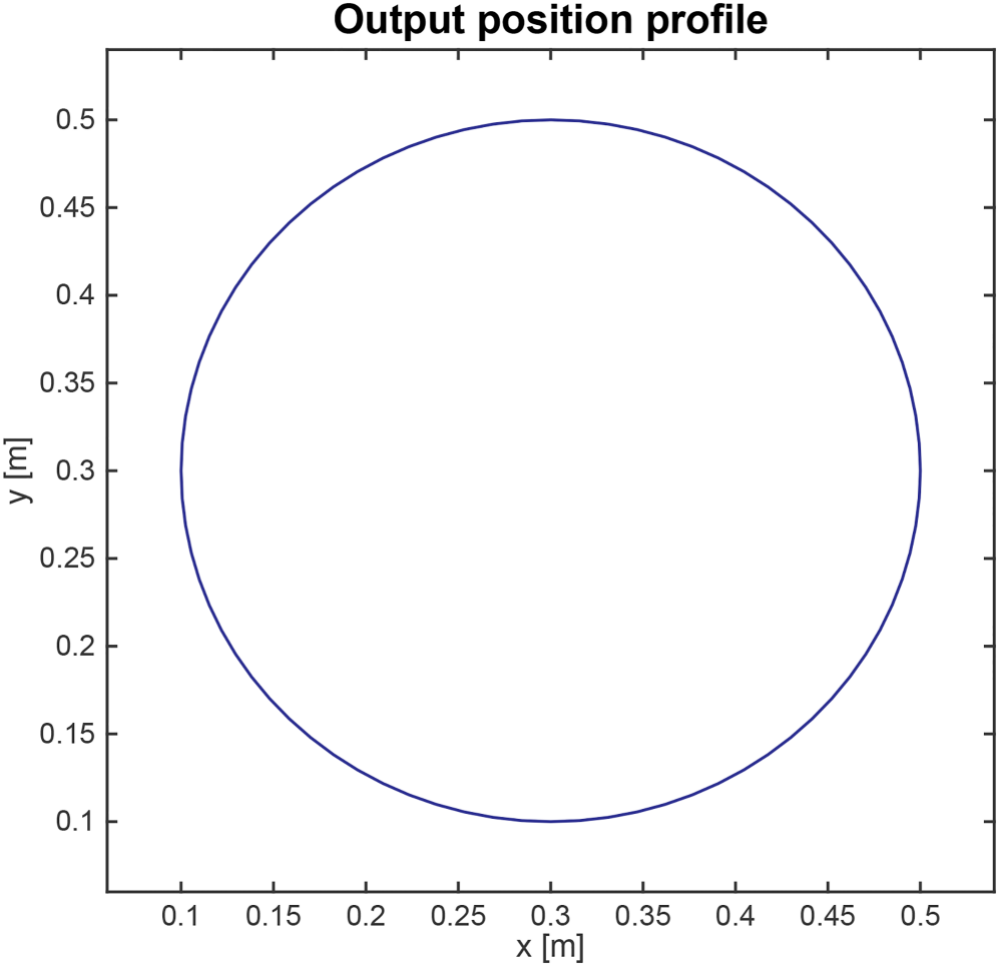
Defined output path for the system.

**Figure 5.**
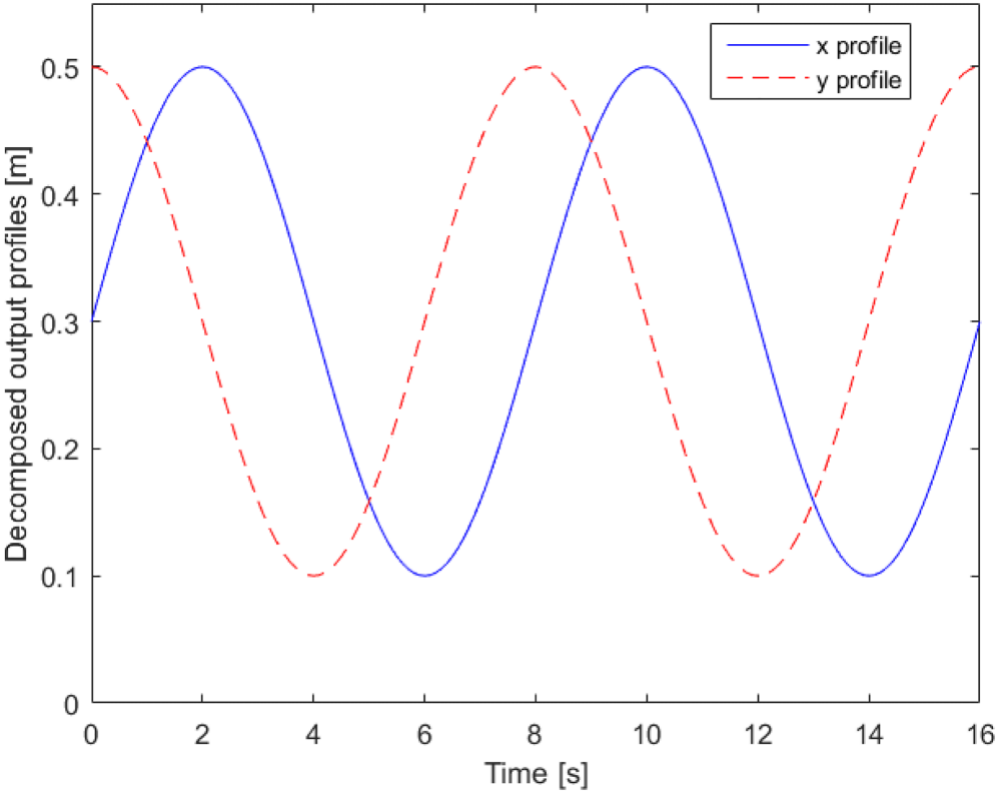
Decomposed output path for the system.

Example 1 For the case study in Figure 2, the desired path of the load component is illustrated in Figure 4, which is a two-dimensional trajectory, hence, $N_{r}$ is 2. The path is decomposed into two output trajectories on the x and y axes as shown in Figure 5.

Definition 1 **(Functional DOF)**

A functional DOF of a component is the translational or rotational axis, denoted as $f_{k\_i}$ , along which component k allows motion. The term i is the number of needed trajectories for that component, that is, iD-trajectory.

Example 2
$f_{1\_1}$ and $f_{2\_1}$ for the two DC motors are $\theta_{1}$ and $\theta_{2}$ . $f_{3\_1}$ and $f_{3\_2}$ and $f_{4\_1}$ and $f_{4\_2}$ for the 2-DOF arm and load components are both x and y .

Definition 2 **(Input and output ports))**

An input/output port is a set of timed signals ( $sig_{i}$ ) of position ( $P_{i}$ ) and force/torque ( $Tq_{i}$ ) at the input/output side of the physical component.

$${sig}_{i}\;:\;[0,T]\to R^{2}$$

$${sig}_{i}(t)=[P_{i}(t),Tq_{i}(t){]}^{T}$$

The sets of input and output ports of component k are $IP_{k}$ and $OP_{k}$ , respectively.

Example 3 As shown in Figure 3, each component model in the case study has input and output ports but there is no component connected to the input ports of DC motors, so the input port sets for these components are empty. The 2-DOF arm is treated as one component which has two input ports and two output ports. Table 1 depicts the detailed information of the input and output ports of each component.

**Table 1.** Input, $ip_{k}^{i}$ , and output ports, $op_{k}^{i}$ . ( k is the component number and i translates to iD-trajectory for that component, if i=1 then it is not mentioned in the index).

	OP	IP
$C_{4}$	$\left\{op_{4}^{1},op_{4}^{2}\right\}$	$\left\{ip_{4}^{1},ip_{4}^{2}\right\}$
	$=\{[\begin{array}{c} x_{l,out},0 \end{array}{]}^{T},$	$=\{[\begin{array}{c} x_{l,in},F_{l,in,x} \end{array}{]}^{T},$
	$\left[\begin{array}{c} y_{l,out},0 \end{array}{]}^{T}\right\}$	$\left[\begin{array}{c} y_{l,in},F_{l,in,y} \end{array}{]}^{T}\right\}$
$C_{3}$	$\left\{op_{3}^{1},op_{3}^{2}\right\}$	$\left\{ip_{3}^{1},ip_{3}^{2}\right\}$
	$=\{[\begin{array}{c} x_{a,out},F_{a,out,x} \end{array}{]}^{T},$	$=\{[\begin{array}{c} \theta_{1},T_{\theta_{1,in}} \end{array}{]}^{T},$
	$\left[\begin{array}{c} y_{a,out},F_{a,out,y} \end{array}{]}^{T}\right\}$	$\left[\begin{array}{c} \theta_{2},T_{\theta_{2,in}} \end{array}{]}^{T}\right\}$
$C_{2}$	$\left\{op_{2}\right\}=\left\{[\begin{array}{c} \phi_{m2},T_{m2,out} \end{array}{]}^{T}\right\}$	∅
$C_{1}$	$\left\{op_{1}\right\}=\left\{[\begin{array}{c} \phi_{m1},T_{m1,out} \end{array}{]}^{T}\right\}$	∅

In Table 1, $\phi_{m1}$ , $T_{m1,out}$ and $\phi_{m2}$ , $T_{m2,out}$ are the output angular position and torques of the first and second DC motors, respectively. $x_{a,out}$ , $F_{a,out,x}$ and $y_{a,out}$ , $F_{a,out,y}$ are the output translational position and forces on the x and y axes for the 2-DOF arm. $\theta_{1}$ , $T_{\theta_{1,in}}$ and $\theta_{2}$ , $T_{\theta_{2,in}}$ are the input angular positions and torques of the arms. $x_{l,out}$ and $y_{l,out}$ are the output position profiles of the load component as the main requirements on the system.

If no external force is applied to the load, then the output force signals of the load are constant 0. $x_{l,in}$ , $F_{l,in,x}$ and $y_{l,in}$ , $F_{l,in,y}$ are the input position and force profiles on the input of the load component.

## Component level concept

### Physical component

The physical dimension ( $Pd_{k}$ ) model of the k th component essentially includes formalized constraints on speed and force/torque using component properties such as size, mass, material, friction or cost. The actual dimensioning of a component is dependent on the system output trajectories and how they are dynamically transferred through the component composition. The physical dimension model of component k consists of design parameters ( $p^{k}$ ), design variables ( $v^{k}$ ), physical design constraints and functions related to them that are defined in Definitions 6 to 8.

Definition 3 **(Actuator component)**

An actuator component is a physical component that provides energy/power/torque to the mechanical system and carries the control signal. Let $\hat{a}$ be the actuator component.

Definition 4 **(Load component)**

The load component is the last component ( K th) in the open-chain system and it carries the position profile requirements for the system and the inertia/mass of the component.

Definition 5 **(Structural component)**

The structural component is a physical component that carries the load and transforms position/torque. Some examples of structural components are robotic arms, transmissions and mechanical springs.

Definition 6 **(Design parameter)**

Design parameters of the physical component model k are real-valued variables

$$p_{i}^{k}\in R,i=1,\ldots ,n_{k}$$

$$p^{k}=[p_{1}^{k},p_{2}^{k},\ldots ,p_{n_{k}}^{k}]\in R^{n_{k}}$$

where $n_{k}$ is the number of design parameters of component k .

Table 2 lists all design parameters of all components of the case study except the material properties of components, which are derived by considering steel material.

**Table 2.** Design parameters.

Design parameters	Value	Physical meaning
$r_{m1}$	0.08	Motor 1 radius (m)
$r_{m2}$	0.077	Motor 2 radius (m)
$rpm_{max}$	20,000	Max speed of the two motors
$r_{1}$	0.04	Arm 1 radius (m)
$r_{2}$	0.02	Arm 2 radius (m)

Definition 7 **(Design variable)**

The design variables ( $v^{k}$ ) of component k are the variables to be determined through the design optimization.

$$v^{k}=[v_{1}^{k},v_{2}^{k},\ldots ,v_{m_{k}}^{k}]$$

$$v_{i}^{k}\in D_{i}^{k}\subseteq R,i=1,\ldots ,m_{k}$$

where the range of $v^{k}$ is

$$D=D_{1}^{k}\times D_{2}^{k}\times \ldots \times D_{m_{k}}^{k}$$

$$v^{k}\in D^{k}\subseteq R^{m_{k}}$$

$m_{k}$ is the number of design variables of component k .

Example 4 The design variables ( $v^{k}$ ) and their ranges for the presented case study are defined logically from a mechanical perspective in Table 3.

**Table 3.** Design variables and defined ranges.

Design variables	Range ( $D_{m}$ )	Physical meaning
$l_{m1}$	[0.081,0.2]	Motor 1 length (m)
$l_{m2}$	[0.077,0.15]	Motor 2 length (m)
$l_{1}$	[0.3,0.35]	Arm 1 length (m)
$l_{2}$	[0.3,0.35]	Arm 2 length m

Definition 8 **(Physical design constraint)**

A physical design constraint ( $cons_{k}$ ) of a component is an equality or inequality constraint related to $p^{k}$ , $v^{k}$ , ${sig}_{i}$ and the properties of the system such as stress, geometrical dimension, deflection and maximum force/torque that the component can handle.

Example 5 The physical constraints include the speed limit, peak torque, root-mean-square (RMS) torque and stress of that component model, which are defined in the physical dimension ( $Pd_{k}$ ) model of each component model and are detailed in the ‘Case study’ section.

Definition 9 **(Design answer)**

The design answers ( $\zeta^{k}$ ) for the k th component are evaluated by using output signals ( $OP_{k}$ ) of that component and $p^{k}$ and $v^{k}$

**text**

$$\zeta^{k}=[\zeta_{1}^{k},\zeta_{2}^{k},\ldots ,\zeta_{Q_{k}}^{k}]$$

$$\zeta_{i}^{k}\in R,i=1,\ldots ,Q_{k}$$

$Q_{k}$ is the number of design answer items for component k , which is related to the number of answers from each component to calculate the overall objective functions of the system optimization, and also for internal calculation purposes to evaluate the physical constraints.

Example 6 Table 4 shows the design answers for each component for the case study.

**Table 4.** Design answers.

Component	Design answers	Physical meaning
DC motor 1	$v_{m1}$	Volume
	$M_{m1}$	Mass
	$J_{m1}$	Inertia
DC motor 2	$v_{m2}$	Volume
	$M_{m2}$	Mass
	$J_{m2}$	Inertia
2-DOF arm	$v_{a}$	Total volume
	$M_{a}$	Total mass
	$m_{1}$	First arm mass
	$m_{2}$	Second arm mass

Definition 10 **(Physical dimension)**

The physical dimension ( $Pd_{k}$ ) model of component k is a function that evaluates the design answers ( $\zeta^{k}$ ) of the model with respect to the $v^{k}$

$$Pd_{k}\;:\;D^{k}\to R^{Q_{k}}$$

where $R^{Q_{k}}$ and $D^{k}$ are the range and domain of function $Pd_{k}$ , respectively. The physical dimension model can be, for example, the defined cost, energy and geometrical models.

Example 7 The physical dimension ( $Pd_{k}$ ) model of each component model is as follows:

1) DC motors:

$$\left[v_{mi},M_{mi},J_{mi}]=Pd_{i}(l_{mi}),(i=1,2\right)$$

2) 2-DOF arm as one component:

$$\left[v_{a},M_{a},m_{1},m_{2}]=Pd_{3}(l_{1},l_{2}\right)$$

The symbols are defined in Table 4.

3) Load component:

The geometrical specifications of the load component are defined by the user. Hence, it includes no $\zeta^{K}$ and $Pd_{K}$ model.

Definition 11 **(Static load transfer model)**

The static load transfer model ( $St_{k}$ ) of component k is a function that transfers the movement ( $P_{i}$ ) and force/torque ( $Tq_{i}$ ) signals defined at the output ports ( $op_{k}$ ) to the profile in the input port (i $p_{k}$ )

$$St_{k}\;:\;D^{k}\times R^{2|OP_{k}|}\to R^{2|IP_{k}|}$$

$$\forall t\in [0,T],ip(t)=St_{k}(v^{k},op(t))$$

Example 8 1) DC motors 1 and 2:

The static load transfer of a component model is applied to its functional DOF. In the case study, both DC motors are fixed statically to an inertial frame, and there is no need for the static load transformation model to be executed since there is no other component defined on the input side of the DC motors. Hence, the input port sets of the two motors are both empty, as illustrated in Table 1.

2) 2-DOF arm:

Static load transfer of the 2-DOF arm is dependent on the design variables ( $v^{3}$ ) and the output profile(s) of the arm itself

$$\left[ip_{3}^{1}(t),ip_{3}^{2}(t)]=St_{3}(v^{3},[op_{3}^{1}(t),op_{3}^{2}(t)]\right)$$

where $ip_{3}^{1}(t)$ is the information on the first input port of the 2-DOF arm in terms of position ( $\theta_{1}$ ) and force ( $T_{\theta 1,in}$ ) and $op_{3}^{1}(t)$ is the information on the output port of the 2-DOF arm, where $y_{a,out}$ and $F_{a,out,y}$ are the position and force information. $v^{3}$ includes the variables $l_{1}$ and $l_{2}$ . The same explanation works also for the second input ( $ip_{3}^{2}(t)$ ) and output ports ( $op_{3}^{2}(t)$ ) of the 2-DOF arm, where ( $\theta_{2}$ ) and ( $T_{\theta 2,in}$ ) are the input position and force information, respectively, and $x_{a,out}$ and $F_{a,out,x}$ are the output position and force information, respectively

$$\begin{array}{r} \left(\theta_{1},\theta_{2},T_{\theta_{1,in}},T_{\theta_{2,in}})=St_{3}(x_{a,out},y_{a,out},F_{a,out,x},F_{a,out,y}\right) \end{array}$$

3) Load component:

The load component is a mass ( $M_{l}$ ) whose static load transfer model is defined as below. There are two position profiles defined on the output of the load component as $x_{l,out}$ and $y_{l,out}$

$$\begin{array}{r} (x_{l,in},y_{l,in},F_{l,in,x},F_{l,in,y})=\\ St_{4}(x_{l,out},y_{l,out},F_{l,out,x},F_{l,out,y}) \end{array}$$

The detailed expressions of the static load transfer models of components for the case study are found in the ‘Static load transformation’ section.

Definition 12 **(Dynamic behaviour model)**

The dynamic behaviour model ( $Dy_{k}$ ) of component k captures the internal dynamics and is applicable to both physical and control components.

Note that the dynamic behaviour of the physical components are described by ordinary differential equations (ODEs). Differential algebraic equations are the interface equations used to configure dynamics in the system modelling and composed system models are as well ODEs. However, stochastic differential equations are not considered in the approach.

Definition 13 **(Flexible component)**

A flexible component composed of two mass/inertia bodies with a constitutive relation. The constitutive relation is an equation that models a subsystem of spring(s) and damper(s).

Definition 14 **(Rigid component)**

A rigid component is just one inertia/mass body. For a rotational body, the inertia is defined in relation to $f_{k\_i}$ of that component.

Example 9 This example distinguishes rigid, flexible and actuator components’ dynamics.

**Figure 6.**
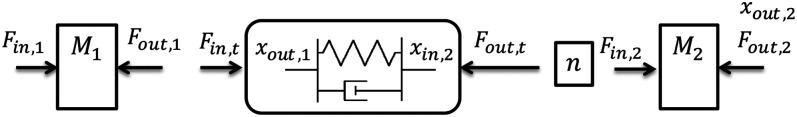
Flexible component.

1) Flexible component:

A flexible component consists of two mass/inertia bodies connected by a spring ( $k_{d}$ ), a damper ( $d_{d}$ ) and possibly a transmission ratio ( n ). For a case including transmission ratio of n≠1 , the torque and position are affected by this ratio as given in (4) and (5). This effect originates from a structure on which the gearbox frame is mounted. The rotational case is corresponding to the transnational case depicted in Figure 6.

(1) $$F_{in,1}-F_{out,1}=M_{1}{\ddot{x}}_{out,1}$$

(2) $$F_{in,2}-F_{out,2}=M_{2}{\ddot{x}}_{out,2}$$

where

(3) $$F_{out,1}=F_{in,t}$$

(4) $$F_{in,2}=n\times F_{out,t}$$

(5) $$x_{out,2}=\frac{x_{in,2}}{n}$$

(6) $$\begin{array}{c} F_{in,t}-F_{out,t}=\\ k_{d}(x_{out,1}-x_{in,2})+d_{d}({\dot{x}}_{out,1}-{\dot{x}}_{in,2}) \end{array}$$

**Figure 7.**
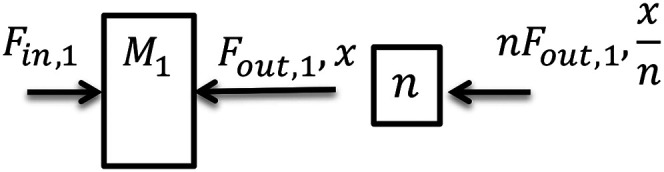
Rigid component.

2) Rigid component:

A rigid component is one mass/inertia and possibly a transmission between the input and output signals as shown in Figure 7 for the translational case.

The dynamics of the rigid body is given in (7):

(7) $$F_{in,1}-\frac{F_{out,1}}{n}=M_{1}\ddot{x}$$

3) Actuator component:

A revolute actuator component as illustrated in Def. 3 has dynamic as given in (8) and (9):

(8) $$T_{in,s}-f(u_{c})=J_{s}\ddot{\phi_{s}}$$

(9) $$f(u_{c})-T_{out,r}=J_{r}\ddot{\phi_{r}}$$

where $f(u_{c})$ represents a function that depends on the control signal $u_{c}$ , which in our case is current or voltage. $J_{s}$ , $\phi_{s}$ and $J_{r}$ , $\phi_{r}$ are the motor’s stator and rotor inertias and rotational angles, respectively. $T_{in,s}$ is the input torque to the stator and $T_{out,r}$ is the output torque of the rotor. In the case of a fixed stator, the motor dynamics reduces to (9).

The detailed expressions related to the dynamic behaviour model and the implemented control method of the case study are described in the ‘Case study’ section.

### Control component

A control component ( $C_{n}$ ) is specified by a parameterized control structure. The control parameters ( $c_{p}$ ) are either given by desired close-loop poles or computed as design variables to satisfy control performance constraints ( $cpc_{\gamma }$ ) such as the max error ( max(er) ) / integrated squared error ( ISE ) or overshoot and response time. To form a closed-loop system, we need to define the actuator and sensor components where the transfer function/state-space model is needed to be formed.

Definition 15 **(Sensor component)**

A sensor component is defined to sense and measure the output variable of a specific physical component. Let ( $\hat{s}$ ) be the notation of this component which has to be specified by the user.

Definition 16 **(Control component)**

The control component represents a control method that is designed to minimize the difference between the desired output profile and actual system output for a particular implementation.

To design the control component, the open-loop plant model must be derived. In this work, the open-loop model is obtained symbolically by Wolfram Mathematica solver. The plant model may be linear or non-linear. In the latter case, the non-linear model can be linearized by the mentioned solver. The detailed approach is represented in Algorithm 1.

Example 10 For the case study, PID control is implemented on the two DOF arms, separately for each degree of freedom, and thus we have six control design parameters of $k_{P1}$ , $k_{D1}$ , $k_{I1}$ , $k_{P2}$ , $k_{D2}$ and $k_{I2}$ . The controlled output trajectories of the system with PID controllers are computed by forward simulation.

Definition 17 **(Control performance constraint)**

The control performance constraint ( cpc ) is a function of $v^{k}$ in the configured physical components, actual output ( $y_{out}(t)$ ) and desired output profile(s) ( $y_{\hat{s}}(t)$ )

$$cpc_{\gamma }(v^{k},y_{out}(t),y_{\hat{s}}(t))\leq b_{\gamma }$$

where b is a limited value that determines the boundary of the cpc and γ is the index number of cpc for the case of more than one performance constraint. The constraints impose requirements on the system’s actual trajectories.

Example 11 Root mean integrated square error ( ISE )^20^ and maximum error ( max(er) ) are defined as the control constraints for the presented case study in Figure 2.

In the case study, the 2-DOF arm is defined as a sensor component, $y_{\hat{s}}(t)$ is the desired angle of each arm ( $\theta_{d1}$ and $\theta_{d2}$ ) and $y_{out}(t)$ is the actual output of the controlled system and is $\theta_{1}$ and $\theta_{2}$ . Therefore, the control performance constraints are as follows:

(10) $$ISE_{i}=\sqrt{\int_{0}^{T}(\theta_{di}(t)-\theta_{i}(t){)}^{2}dt},(i=1,2)$$

(11) $$max(er_{i})=max(|\theta_{di}(t)-\theta_{i}(t)|),(i=1,2)$$

## Dynamic system modelling

For dynamic system modelling, the physical and control components are connected into a graph called the IDIOM model.

Definition 18 **(IDIOM model)**

The IDIOM model ( $G_{p}$ ) is a system configuration that consists of IDIOM objects as vertices, that is, physical ( $C_{k}$ ) and control components ( $C_{n}$ ) and IDIOM connectors as edges ( E ), that is, the interfaces where each is connecting two components

$$G_{p}=(C_{1},E_{1,2},C_{2},\ldots ,C_{K-1},E_{K-1,K},C_{K})$$

Each edge is an IDIOM connector which is normally an arrow. There is an arrow between the objects k and k−1 for k=2,…,K , which carries information as movement ( $P_{i}(t)$ ) and force/torque ( $Tq_{i}(t)$ ). The arrow $E_{k-1,k}$ is a pre-defined symbolic interface equation, which specifies that the signals on the output port ( $op_{k-1}$ ) is equal to signals on in the input port ( $ip_{k}$ )

$$(op_{k-1},ip_{k})\in OP_{k-1}\times IP_{k}$$

$$E_{k-1,k}\;:\;\left\{ \begin{array}{c} op_{k-1}.(P_{i}(t))=ip_{k}.(P_{i}(t))\\ op_{k-1}\cdot Tq_{i}(t)=ip_{k}\cdot Tq_{i}(t) \end{array}\right.$$

Theorem 1 Given a set of physical components $\overset{K}{\underset{k=1}{⨂}}C_{k}$ as IDIOM objects allocated to a composition, there exists arrow(s) as $E_{1,2},\ldots ,E_{K-1,K}$ representing IDIOM connector interface to ensure an IDIOM model ( $G_{p}$ ).

Theorem 2 Given a composition $G_{p}$ ,

*(i) the vertices and edges are invariant,*

*hence, symbolic representation of $St_{k}$ and $Dy_{k}$ are invariant.*

(ii) $N_{r}$ , $p^{k}$ , $v^{k}$ and b are variant,

hence, numerical evaluation of **$\zeta^{k}$** and $cpc_{i}$ are variant.

**Figure 8.**
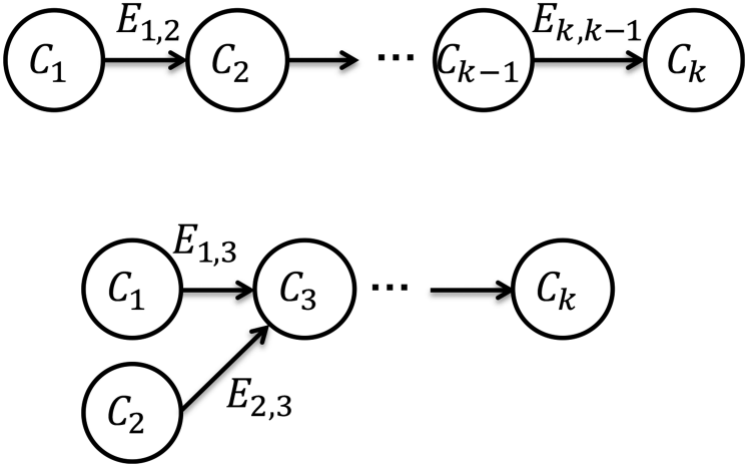
Two system compositions of components.

Figure 8 shows the open-chain component composition of a system in IDIOM. Currently, the delimitation of the framework is that it only handles systems composed as open-chain configurations unless a control component is included.

The component models $Pd_{k}$ and $Dy_{k}$ as described in Definitions 10 and 12 are only used in the component level.

Example 12 We use the case study shown in Figure 2 as an example of how an IDIOM model $G_{p}$ is configured. There are three connector interfaces $E_{1,3}$ , $E_{2,3}$ and $E_{3,4}$ , which connect $C_{1}$ (DC motor 1) and $C_{3}$ (2-DOF), $C_{2}$ (DC motor 2) and $C_{3}$ (2-DOF), $C_{3}$ (2-DOF arm) and $C_{4}$ (load component), respectively.

1) DC motor 1 and 2-DOF arm

(12) $$\begin{array}{c} E_{1,3}\;:\\ \phi_{m1}=\theta_{1}\\ T_{m1,out}=T_{\theta 1,in} \end{array}$$

2) DC motor 2 and 2-DOF arm

(13) $$\begin{array}{c} E_{2,3}\;:\\ \phi_{m2}=\theta_{2}\\ T_{m2,out}=T_{\theta 2,in} \end{array}$$

3) 2-DOF arm and load

(14) $$\begin{array}{c} E_{3,4}\;:\\ x_{a,out}=x_{l,in}\\ F_{a,out,x}=F_{l,in,x}\\ y_{a,out}=y_{l,in}\\ F_{a,out,y}=F_{l,in,y} \end{array}$$

## Optimization

Definition 19 **(IDIOM objective function)**

The IDIOM objective function ( f ) is a function which is based on **$\zeta^{k}$** of each component’s $Pd_{k}$ , $St_{k}$ and $Dy_{k}$ model, and $cpc_{\gamma }$ of the entire system:

$$f_{\chi }\;:\;D_{1}\times D_{2}\times \cdots \times D_{k}\to R$$

$$\min_{v^{k}\in D^{k}}\sum_{\;j=1}^{\chi }f_{j}(v^{k})$$

$$s.t.\left\{ \begin{array}{c} \zeta^{k}=Pd_{k}(v^{k})\\ ip_{k}(t)=St_{k}(v^{k},op_{k}(t))\\ op_{k-1}(i)=ip_{k}(i),i\in 2|N_{r}|\\ cpc_{i}\leq b,i=1,\ldots ,\gamma \end{array}\right.$$

χ is the number of the objective functions.

The objective functions are based on pre-specified component cost functions (e.g., mass, cost, and energy loss) and each function in each component model may have multiple outputs and they together form **$\zeta^{k}$** in Definition 9. For example, the ‘design’ function may return volume, length and mass. Some of these functions are already added to the component models, and additional functions may be defined based on each component behaviour and properties.

Example 13 With the given physical constraints, the control performance constraints $b_{\gamma }$ and the component variables and parameters ( $v^{k}$ and $p^{k}$ ) in Tables 2, 3 and 5 (four $v^{k}$ , six control design variables and five ${\;p}^{k}$ ), the objective function can be resolved. For the case study, the objective is to minimize the volume of the entire system:

(15) $$\begin{array}{rl} \min_{v^{k}\in D^{k}}\sum_{\;j=1}^{\chi }f_{j}(v^{k})= & \min_{l_{m1},l_{m2},l_{1},l_{2}\in D^{4}}(\pi r_{m1}^{2}l_{m1}\\ & +\pi r_{m2}^{2}l_{m2}+\pi r_{1}^{2}l_{1}+\pi r_{2}^{2}l_{2}) \end{array}$$

**Table 5.** Control design variables.

Control parameters	Range
$k_{P1}$	[10,200]
$k_{D1}$	[4,50]
$k_{I1}$	[1,50]
$k_{P2}$	[10,200]
$k_{D2}$	[4,50]
$k_{I2}$	[1,50]

An integrated control and physical design optimization approach is used in the IDIOM framework to emphasize the interdependent relation between physical and dynamic design. The optimizer implemented in the IDIOM framework is a genetic algorithm (GA). GA is chosen since it can handle problems with discrete design variables and is implementable on problems with non-linear objectives. GA is an approach to solve both constrained and unconstrained problems and can be applied to a diverse range of complex problems. To use other optimization algorithms with the method, the corresponding algorithm should be integrated into the method and implemented to the IDIOM framework. The purpose of holistic design optimization is to achieve the best overall system properties rather than design optimization of each component independently. Static properties, physical limitations and control constraints are evaluated concurrently by the optimization solver, where the ‘physical dimension’ models are also calculated. In previous work by Frede et al.,^2^ the use of multiple optimization criteria is enabled. Maximum generation number and population size are to be defined by the user, if not; the default values are 200 and 40, respectively.

## System model computation

**Figure 9.**
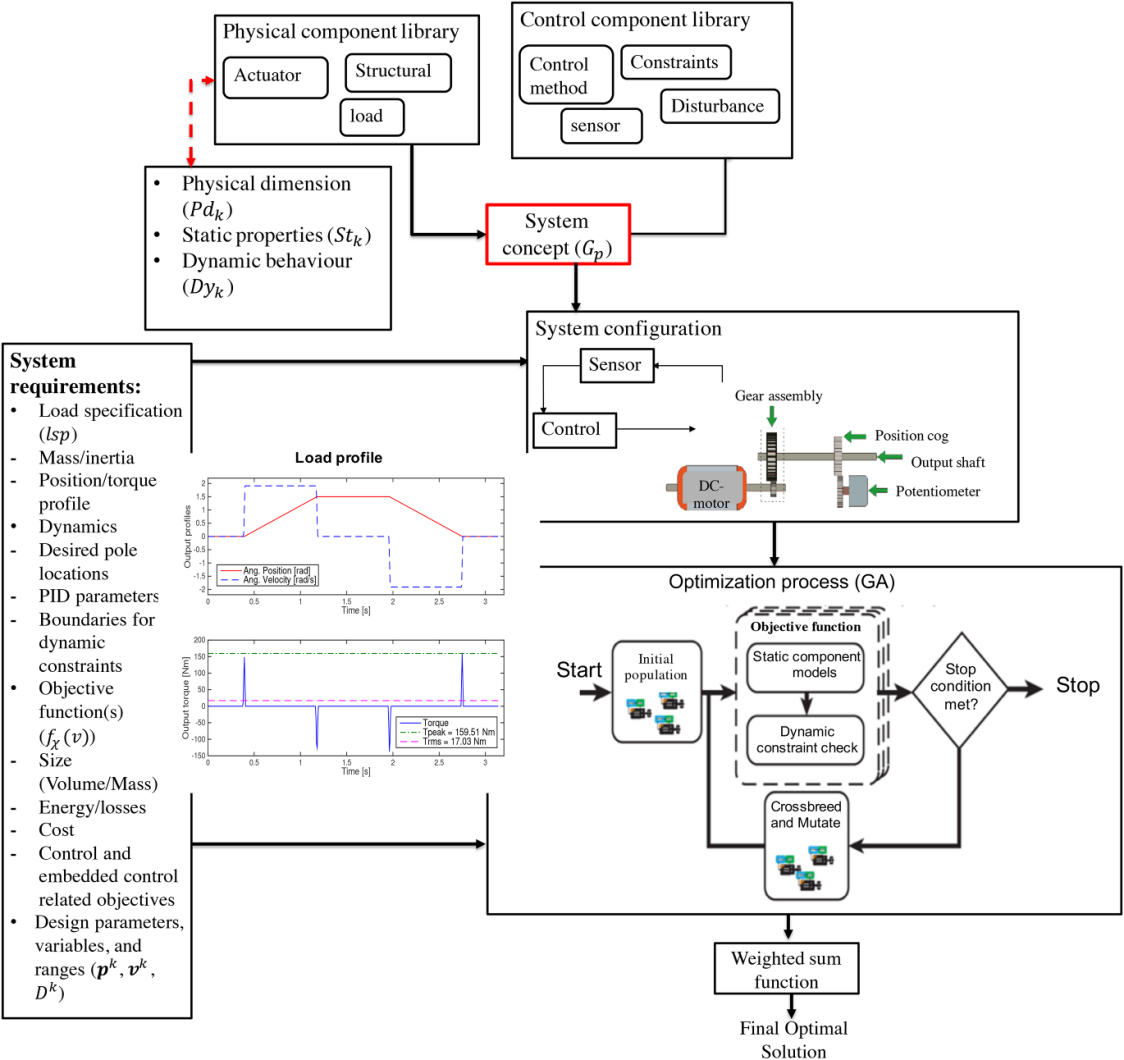
Overview of the methodology.

The mechatronic system concept is configured using a physical and dynamic component library based on detailed specifications from the user. The methodology implemented in the framework with all the constituent components and models is shown in Figure 9. The system concept is generated by drag and drop of physical and control components from the component library of the framework. After having a decision on a system configuration and requirements in terms of load specification, required dynamic performance and optimization objectives, the system is optimized according to Algorithms 1 to 3. It should be noted that all the system models implemented in the framework so far, including the required motion profiles, are continuous time.

For an arbitrary system composition with K component models, with an objective function $f_{\chi }(x)$ , the model evaluation, component dimensioning and optimization goes through the following three algorithms. Algorithm 1 builds the dynamic configuration of the component models. This means that linear or non-linear dynamic differential equations of each component together with the connector interfaces are gathered as described in Defintion 18 to construct the open-loop and closed-loop models in terms of transfer functions or state-space models.

The inputs for Algorithm 1 are the system concept as a graph ( $G_{p}$ ) described in the ‘Dynamic system modelling’ section, the system attributes for the control method such as actuator component ( $\hat{a}$ ), sensor component ( $\hat{s}$ ) and the control parameters ( $c_{p}$ ), which are presented in the ‘Basics of the supported software framework’ and ‘Component level concept’ sections. The outputs of Algorithm 1 are the symbolic representation of the open-loop and closed-loop ( $G_{cl}$ ) system models in terms of transfer functions/state space models.

The algorithm is executed in the chain between the two specified physical components as $\hat{a}$ and $\hat{s}$ , see Algorithm 1 pseudo code. From lines 3 to 10, $Dy_{k}$ (Definition 12) of each physical component model from $\hat{a}$ to $\hat{s}$ is read and written in a newly formed equations list ( Eq ), which is empty in the beginning of the algorithm. The interface connectors between physical components presented in Definition 18 are added to the Eq if and only if $k\neq \hat{s}$ . Hence, Eq is completely formed when $k=\hat{s}$ .

One solution to solve symbolic algebraic differential equations is to use the software Wolfram Mathematica. In lines 11 to 13 in Algorithm 1, the Mathematica kernel is started and the symbolic Eq is sent from Matlab to Mathematica together with the control parameters ( $c_{p}$ ) and the requirements on the input/output variables of the transfer function and input/output/state variables of the state space model. The model is derived in Mathematica and is sent back to Matlab. The control component ( $C_{n}$ ) with the control method and defined $c_{p}$ is integrated with the open-loop model ( $G_{\;p}$ ) to form the closed-loop model ( $C_{cl}$ ). In case the control method is defined by a simple transfer function for instance a PID, a proportional–derivative or a proportional–integral control method, then the closed-loop system can be derived using (16) and are outputted in line 16 in a form of transfer function/state-space model.

(16) $$C_{cl}=\frac{C_{n}\cdot TF_{op}}{1+C_{n}\cdot TF_{op}}$$

**Algorithm 1** Dynamic System Configuration (DSC)
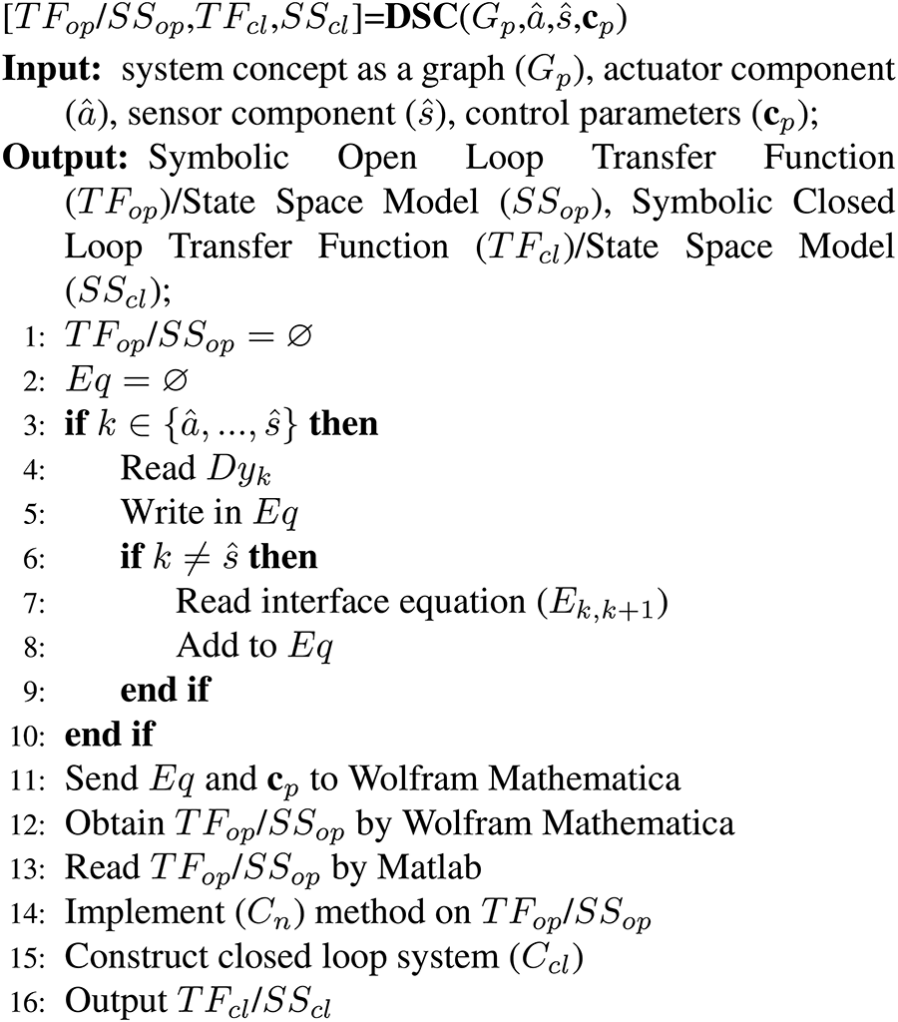

Proof According to Algorithm 1 and Definition 18, for an arbitrary IDIOM model composition of $G_{p}$ with k=1,…,K vertices, there exists an edge $E_{k,k+1}$ between the two vertexes of k and k+1 if and only if k≠K . Using $E_{k,k+1}$ and $Dy_{k}$ for each component of $k\in \left\{\hat{a},\ldots ,\hat{s}\right\}$ , a monotone algebraic dynamic system will be generated, which will be referred as an open loop system model (transfer function ( $TF_{op}$ ) or state space model ( $SS_{op}$ )). This follows that there exists an edge-labelled, directed graph with vertices $C_{\hat{a}},\ldots ,C_{\hat{s}}$ and edges $E_{\hat{a},\hat{a}+1},\ldots ,E_{\hat{s}-1,\hat{s}}$ .  □

Proof According to Algorithm 1, the dynamic system composition in the IDIOM framework is provable, so it is complete.  □

Algorithm 2 uses physical dimension ( $Pd_{k}$ ) and static load transfer ( $St_{k}$ ) models of each component to determine the size/energy/cost of that component as its contribution to the overall objective function(s). The design answers (** $\zeta^{k}$ **) of each physical component is derived to further examine the feasibility of the physical design for the system.

The inputs for Algorithm 1 are the system concept in a graph ( $G_{p}$ ), the number of requirements ( $N_{r}$ ), output profile ( $op_{K}(t)$ ) of the load component ( K ), design parameters ( $p^{k}$ ), design variables ( $v^{k}$ ) and their ranges ( $D^{k}$ ), and the physical constraints, which are defined in Definition 2 and Definitions 4 to 8. The output is the design answers (** $\zeta^{k}$ **) of each physical component in terms of its dimensioning and is defined in Definition 9.

In lines 1–3, the load component’s ( $C_{K}$ ) output profile ( $op_{K}(t)$ ) is called and the static load transfer function ( $St_{K}$ ) is executed on it to calculate the input profile ( $ip_{K}(t)$ ) of the same component. The load component itself does not include a physical dimension model ( $Pd_{K}$ ), therefore, no-load transfer computation is needed.

In the next step, in line 4, a loop starts to go through the components from output to input side for the algorithm to execute the physical dimension ( $Pd_{k}$ ) model on the physical components using the output profile ( $op_{k}(t)$ ). Hence, the loop starts from component K−1 and ends at the first component, which is generally the actuator component and the interface connectors are applied (line 5), that is, the profiles on the input side of $C_{k}$ are defined to be equal to the output of $C_{k-1}$ . In lines 6–17 in the same loop, the $Pd_{k}$ model is computed using $p^{k}$ and $v^{k}$ and component k is designed while adhering to the physical design constraints ( $cons_{k}$ ) in Definition 8 to calculate the design answers ( ${As}^{k}$ ) in the component level for physical scaling and dimensioning. Unless the component is the first component (actuator component) in the system chain, the static load transfer model ( $St_{k}$ ) presented in Definition 11 is executed to calculate the input profile ( $ip_{k}$ ) of the same component using its output port information introduced in Definition 2. If the physical design constraints are satisfied, the design answers (** $\zeta^{k}$ **) are calculated and outputted otherwise the design answers are empty (** $\zeta^{k}$** =∅ ). The algorithm is completed when **$\zeta^{k}$** for k=1,…,K−1 are evaluated.

**Algorithm 2** Component Level Static Model Evaluation (CLSME)
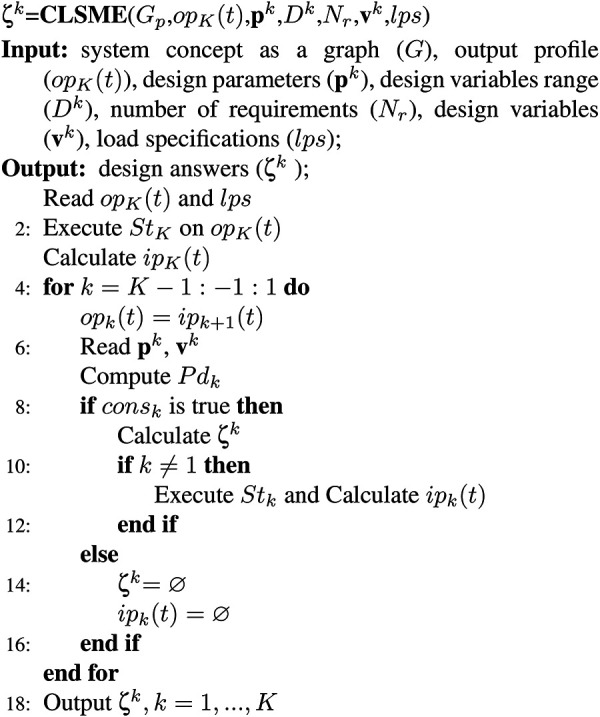


Algorithm 3 checks for the satisfaction of the control constraints ( $cpc_{\gamma }$ ) and computes the optimization objectives for the system of physically designed components. In line 2, Algorithm 2 is executed to get the design answers (** $\zeta^{k}$ **) of the particular system composition. In line 3, Algorithm 1 is executed such that the symbolic transfer function ( $TF_{cl}$ ) or state space model ( $SS_{cl}$ ) are computed. If the design answers (** $\zeta^{k}$** ,k=1,…,K−1 ) from Algorithm 2 are real values then the local optimum objective is computed by re-using (** $\zeta^{k}$ **) from Algorithm 2, otherwise the local optimum is invalid. The numerical values of $TF_{cl}$ or $SS_{cl}$ are evaluated in lines 5 and 6. In lines 7–14, the control constraints ( $cpc_{\gamma }$ ) are calculated for γ number of constraints and their satisfaction are checked to be in the defined b boundary. The algorithm is repeated until the optimization generation and population size is finished and the global optimized objective ( $\min_{v^{k}\in D^{k}}\sum_{\;j=1}^{\chi }f_{j}(v^{k})$ ) is evaluated in line 20 and the optimizer terminates with an optimal design and control result for the IDIOM model.


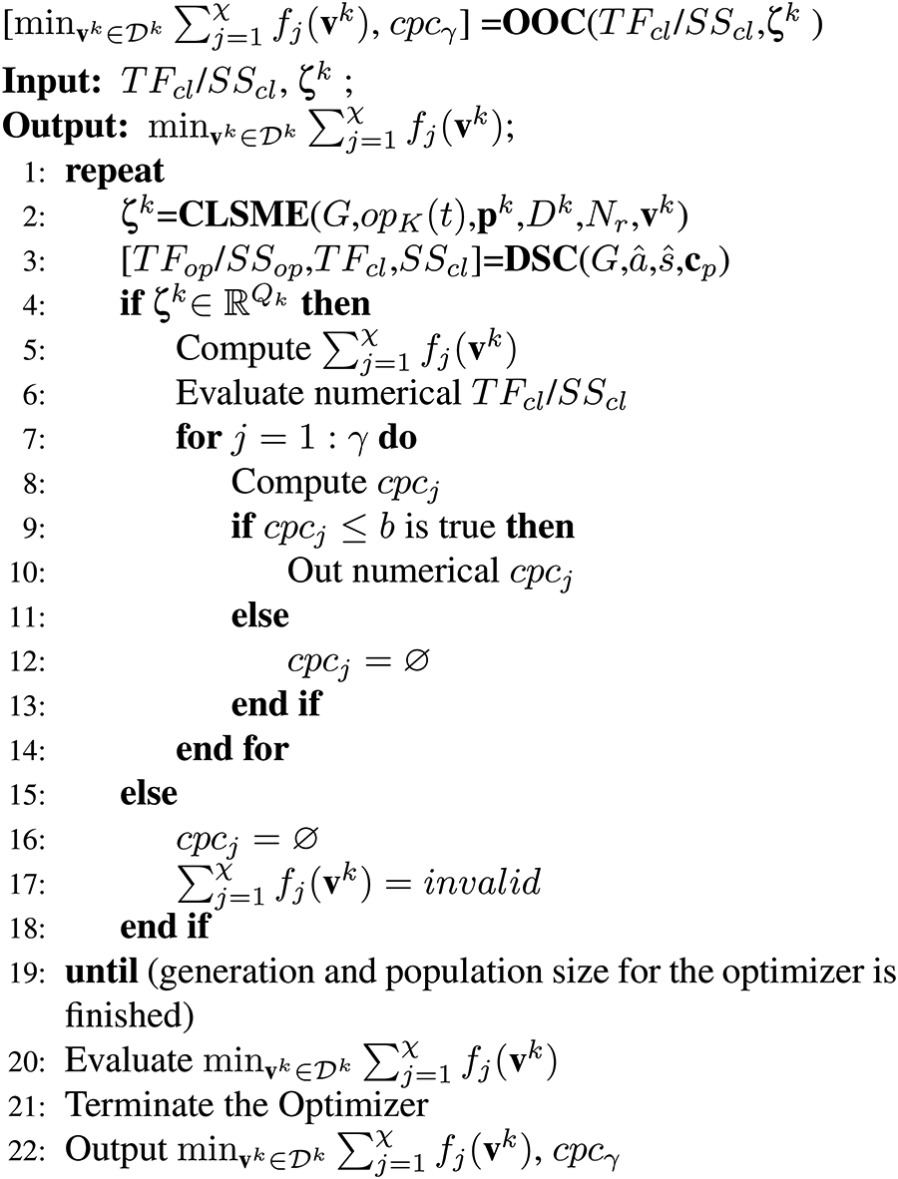

Proof Given a composition of components as an IDIOM model (Definition 18), using the method presented in Algorithms 1 to 3 there is a consistent optimization solution independent of the solver, if and only if $p^{k}$ , D , $N_{r}$ and $OP_{K}$ for the physical components and $c_{p}$ and b in $cpc_{\gamma }$ for the control component are defined consistent. This follows for every composition, hence, the IDIOM model is sound.  □

## Case study

### Physical design constraints

The physical design constraints (Definition 8) of each case study components (Figure 2) are as follows:

1) Physical constraints on the motor model:

The physical design constraints of the two DC motors are adopted from scaling approaches presented by Roos et al.,^22,23^ which specify the relationship between the rated torque and the actual RMS torque as in (17):

(17) $$T_{m,rated}\geq T_{rms}$$

where $T_{m,rated}$ and $T_{rms}$ are the rated torque, and the RMS torque, respectively. The rated torque is derived based on the mechanical, magnetic and thermal effects^22^:

(18) $$T_{m,rated}=C_{m}l_{m}r_{m}^{2.5}$$

where $C_{m}$ is a motor type constant for the same cooling conditions, $l_{m}$ is the motor’s rotor length and $r_{m}$ is the radius of the stator. The motor’s RMS torque is derived as

(19) $$T_{rms}=\sqrt{\frac{1}{\tau }\int_{0}^{\tau }((C_{mj}l_{m}r_{m}^{4}+J_{m}){\ddot{\phi }}_{m,out}+T_{m,out}{)}^{2}dt}$$

where $C_{mj}$ is a constant for a specific machine type and is derived from a reference motor of the same type, τ is the cycle time of the output profile, that is, the time period which the output profile is valid, ${\ddot{\phi }}_{m,out}$ is the angular acceleration of the rotor. $T_{m,out}$ is the output torque of the motor and $J_{m}$ is the motor inertia. Combining (17) to (19) results in (20):

(20) $$\begin{array}{c} C_{m}l_{m}r_{m}^{2.5}\geq \sqrt{\frac{1}{\tau }\int_{0}^{\tau }((C_{mj}l_{m}r_{m}^{4}+J_{m}){\ddot{\phi }}_{m,out}+T_{m,out}{)}^{2}dt} \end{array}$$

**Figure 10:**
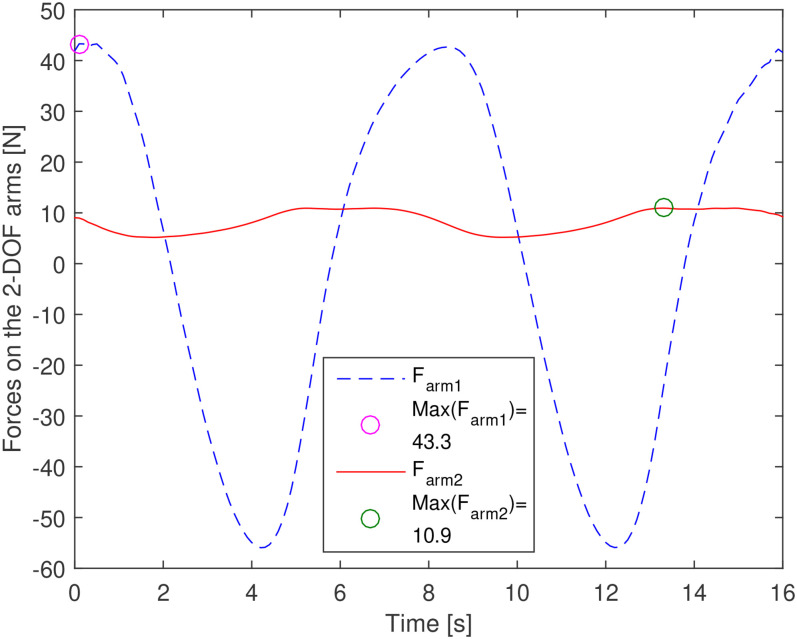
Forces affecting the 2-DOF arm and the max values of them.

2) Physical constraints on the 2-DOF arm:

The constraint on the 2-DOF arm is that the strength of the two arms is larger than the required force. The equation is derived from Hamrock et al.^24^:

(21) $$max\left(\frac{F}{A}\right)\leq \tau_{m}$$

where F is the force orthogonal to the cross-sectional area and is derived from the output force signals on the 2-DOF arm, A is the cross-sectional area and $\tau_{m}$ is the maximal stress of the arm material (see Figure 10).

### Static load transformation

1) $St_{k}$ of the motor models:

There is no need to execute $St_{k}$ for the motor models since there is no other component that needs dimensioning on the input of the motor.

2) $St_{k}$ of the 2-DOF arm:

The $St_{k}$ expressions (Definition 11) for each component model k of the case study (Figure 2) are as follows.

The angular positions of the arms are derived from inverse kinematics.^25^ For the angular position of the second arm ( $\theta_{2}$ ), we have

(22) $$\theta_{2}=\cos^{-1}\left(\frac{x_{a,out}^{2}+y_{a,out}^{2}-l_{1}^{2}-l_{2}^{2}}{2l_{1}l_{2}}\right)$$

For the first arm’s angular position ( $\theta_{1}$ ), we have

(23) $$\theta_{1}=\tan^{-1}\left(\frac{y_{a,out}}{x_{a,out}}\right)-\tan^{-1}\left(\frac{l_{2}\sin\;(\theta_{2})}{l_{1}+l_{2}\cos\;(\theta_{2})}\right)$$

We employ Lagrangian mechanics to derive the relation between the input torque and the output position. Let $x_{i}$ , $y_{i}$ ( i=1,2 ) be the position coordinates of the tips of the two arms as illustrated in Figure 2.

(24) $$x_{1}=l_{1}\cos\;(\theta_{1}),x_{2}=l_{1}\cos\;(\theta_{1})+l_{2}\cos\;(\theta_{1}+\theta_{2})$$

(25) $$y_{1}=l_{1}\sin\;(\theta_{1}),y_{2}=l_{1}\sin\;(\theta_{1})+l_{2}\sin\;(\theta_{1}+\theta_{2})$$

The kinetic and potential energies of the two arms considering the mass of each arm as a point at the tip are derived from Okubanjo et al.^26^

The Lagrangian of the robot arm is taken from Efe^27^ and Mahil and Al-Durra,^28^ which are used to evaluate system dynamics.

**Figure 11:**
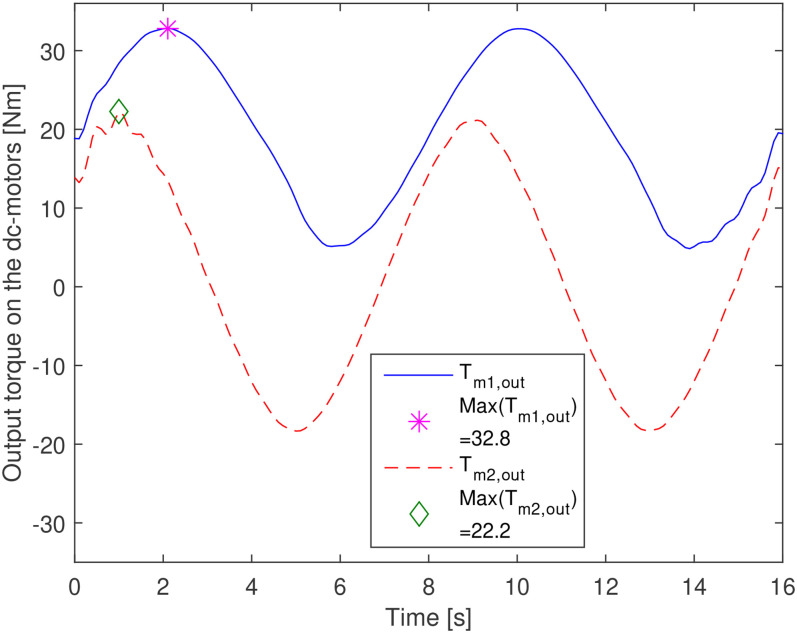
Torques and their maximum values on the DC motors.

Later in the design, $St_{k}$ considers the maximal values of the torque/force throughout the chain of components and use them in the physical dimensioning of each component. Figure 11 shows the maximal output torques of the two DC motors, which are equal to the input torques of the 2-DOF arm.

3) $St_{k}$ on the load component:

(26) $$M_{l}{\ddot{x}}_{l,in}=F_{l,in,x}-F_{l,out,x}$$

(27) $$x_{l,in}=x_{l,out}$$

(28) $$M_{l}{\ddot{y}}_{l,in}=F_{l,in,y}-F_{l,out,y}$$

(29) $$y_{l,in}=y_{l,out}$$

(30) $$F_{l,out,x}=F_{l,out,y}=0$$

where ${\ddot{x}}_{l,in}$ and ${\ddot{y}}_{l,in}$ are the accelerations derived from the two position profiles ( $x_{l,out}$ and $y_{l,out}$ ). $F_{l,in,x}$ , $F_{l,in,y}$ and $F_{l,out,x}$ , $F_{l,out,y}$ are the input and output forces in the x and y directions.

### Dynamic behaviour model

The detailed $Dy_{k}$ ^21^ for each component model k in the case study (Figure 2) are as follows:

1) DC motor:

For both of the DC motors we have

(31) $$K_{Tj}\cdot i_{j}=T_{mj,out}+J_{mj}{\ddot{\phi }}_{mj},(j=1,2)$$

where $K_{Tj}$ is the torque constant dependent on the motor type and the reference motor according to the scaling approach presented by Roos,^23^ $i_{j}$ is the current which is considered as actuation/control signal acting on the dynamic input port. $T_{mj,out}$ and ${\ddot{\phi }}_{mj}$ are torque and angular acceleration of the DC motor and $J_{mj}$ is the total rotor inertia.

2) 2-DOF arm:

We employ Lagrangian mechanics^27,28^ to evaluate the dynamic differential equations.

3) Load:

The load component’s dynamics is approximated by a point mass whose dynamics at the x and y axes is given in (26) to (30).

For evaluating the system dynamics, two PID controllers are employed on the 2-DOF arm, where the currents $i_{1}$ and $i_{2}$ are the control signals and $\theta_{1}$ and $\theta_{2}$ are the 2-DOF arm angles as the measured positions. Combining the dynamic equations elaborated earlier, we derive the state-space model of the system as

(32) $$\ddot{\theta }=M(\theta {)}^{-1}(-C(\dot{\theta },\theta )\dot{\theta }-G(\theta )+T_{\theta ,in})$$

where M is the inertia matrix in the 2-DOF arm dynamics and the dimension is 2×2 , C is a 2×2 Coriolis matrix, G is a 2×1 gravity matrix, $T_{\theta ,in}$ ( 2×1 ) is the input torques of the 2-DOF arm, which are equal to the output torques ( $T_{m,out}$ ) of the DC motors, θ ( 2×1 ) is the angular positions of the 2-DOF arm

(33) $$T_{\theta j,in}=T_{mj,out},\;(j=1,2)$$

(34) $$\begin{array}{c} \left[\begin{array}{cc} m_{11} & m_{12}\\ m_{21} & m_{22} \end{array}\right]\left[\begin{array}{c} {\ddot{\theta }}_{1}\\ {\ddot{\theta }}_{2} \end{array}\right]=-\left[\begin{array}{cc} c_{11} & c_{12}\\ c_{21} & c_{22} \end{array}\right]\left[\begin{array}{c} {\dot{\theta }}_{1}\\ {\dot{\theta }}_{2} \end{array}\right]\\ -\left[\begin{array}{c} G_{11}\\ G_{21} \end{array}\right]+\left[\begin{array}{c} K_{T1}i_{1}-J_{m1}{\ddot{\phi }}_{m1}\\ K_{T2}i_{2}-J_{m2}{\ddot{\phi }}_{m2} \end{array}\right] \end{array}$$

and we have (12) and (13)

(35) $$\left[\begin{array}{c} {\ddot{\phi }}_{m1}\\ {\ddot{\phi }}_{m2} \end{array}\right]=\left[\begin{array}{c} {\ddot{\theta }}_{1}\\ {\ddot{\theta }}_{2} \end{array}\right]$$

Comparing Lagrangian mechanics^27,28^ for the 2-DOF arm with (34), we derive elements of M(θ) , $C(\dot{\theta },\theta )$ and G(θ) matrices^29^:

(36) $$\begin{array}{c} m_{11}=m_{1}l_{1}^{2}+m_{2}l_{1}^{2}+m_{2}l_{2}^{2}+2m_{2}l_{1}l_{2}\cos\;\theta_{2}+J_{m1}\\ m_{12}=m_{2}l_{2}^{2}+m_{2}l_{1}l_{2}\cos\;\theta_{2}\\ m_{21}=m_{2}l_{2}^{2}+2m_{2}l_{1}l_{2}\cos\;\theta_{2}\\ m_{22}=m_{2}l_{2}^{2}+J_{m2} \end{array}$$

(37) $$\begin{array}{c} c_{11}=-2m_{2}{\dot{\theta }}_{2}l_{1}l_{2}\sin\;\theta_{2}\\ c_{12}=-m_{2}{\dot{\theta }}_{2}l_{1}l_{2}\sin\;\theta_{2}\\ c_{21}=-m_{2}{\dot{\theta }}_{1}l_{1}l_{2}\sin\;\theta_{2}\\ c_{22}=0 \end{array}$$

(38) $$\begin{array}{c} G_{11}=m_{1}gl_{1}\cos\;\theta_{1}+m_{2}gl_{1}\cos\;\theta_{1}+m_{2}gl_{2}\cos\;(\theta_{1}+\theta_{2})\\ G_{21}=m_{2}gl_{2}\cos\;(\theta_{1}+\theta_{2}) \end{array}$$

The control input vector is

(39) $$\left[\begin{array}{c} K_{T1}i_{1}\\ K_{T2}i_{2} \end{array}\right]=\left[\begin{array}{c} {\hat{\tau }}_{1}\\ {\hat{\tau }}_{2} \end{array}\right]$$

By substituting (39) in (34), we have the controlled system equation as follows:

(40) $$\begin{array}{c} \left[\begin{array}{c} {\ddot{\theta }}_{1}\\ {\ddot{\theta }}_{2} \end{array}\right]=\hat{M}(\theta_{1},\theta_{2}{)}^{-1}(-C({\dot{\theta }}_{1},\theta_{1},{\dot{\theta }}_{2},\theta_{2})\left[\begin{array}{c} {\dot{\theta }}_{1}\\ {\dot{\theta }}_{2} \end{array}\right]-G(\theta_{1},\theta_{2})+\left[\begin{array}{c} {\hat{\tau }}_{1}\\ {\hat{\tau }}_{2} \end{array}\right]) \end{array}$$

where input ${\hat{\tau }}_{1}$ and ${\hat{\tau }}_{2}$ to the system are the outputs of the PID control method as follows:

(41) $$\begin{array}{c} \left[\begin{array}{c} {\hat{\tau }}_{1}\\ {\hat{\tau }}_{2} \end{array}\right]=\left[\begin{array}{c} k_{P1}(\theta_{d1}-\theta_{1})\\ k_{P2}(\theta_{d2}-\theta_{2}) \end{array}\right]-\left[\begin{array}{c} k_{D1}{\dot{\theta }}_{1}\\ k_{D2}{\dot{\theta }}_{2} \end{array}\right]+\left[\begin{array}{c} k_{I1}\int e(\theta_{1})dt\\ k_{I2}\int e(\theta_{2})dt \end{array}\right] \end{array}$$

$\theta_{d1}$ and $\theta_{d2}$ are defined as desired positions and $\theta_{1}$ and $\theta_{2}$ are the actual controlled outputs ( $y_{out}(t)$ ).

By substituting (41) in (40), we obtain

(42) $$\begin{array}{rl} \left[\begin{array}{c} {\ddot{\theta }}_{1}\\ {\ddot{\theta }}_{2} \end{array}\right]= & \;M(\theta_{1},\theta_{2}{)}^{-1}(-C({\dot{\theta }}_{1},\theta_{1},{\dot{\theta }}_{2},\theta_{2})\left[\begin{array}{c} {\dot{\theta }}_{1}\\ {\dot{\theta }}_{2} \end{array}\right]\\ & -G(\theta_{1},\theta_{2})+\left[\begin{array}{c} k_{P1}(\theta_{d1}-\theta_{1})\\ k_{P2}(\theta_{d2}-\theta_{2}) \end{array}\right]\\ & -\left[\begin{array}{c} k_{D1}{\dot{\theta }}_{1}\\ k_{D2}{\dot{\theta }}_{2} \end{array}\right]+\left[\begin{array}{c} k_{I1}\int e(\theta_{1})dt\\ k_{I2}\int e(\theta_{2})dt \end{array}\right]) \end{array}$$

The parameters of the two PID controllers are determined by design optimization. The ranges of the controller parameters are estimated by trial and error. Smaller ranges are beneficial for reducing the computation time of design optimization. The ranges are given in Table 5, the control method parameters should satisfy the control constraints.

## Results and discussion

Using an optimal PID control, the required trajectory tracking performance is achieved and the optimal system is designed. The result of the optimization for two design problems is presented and compared in Table 6 where we altered only the control constraints boundaries in problems 1 and 2 to check the effect of the constraint on the physical design of the system. The boundaries for the control constraints of ISE and max(er) are defined as follows: Problem 1:

ISE<0.02

max(er)<0.04

**Table 6.** Optimization output.

Optimization parameters	Problem 1	Problem 2
$l_{m1}$	107 mm	112 mm
$l_{m2}$	96 mm	78.2 mm
$l_{1}$	342 mm	308.8 mm
$l_{2}$	311.5	326.6 mm
v	$0.0082\;m^{3}$	$0.0076\;m^{3}$
$k_{P1}$	197	200
$k_{I1}$	1	50
$k_{D1}$	4	4
$k_{P2}$	125	200
$k_{I2}$	35	38
$k_{D2}$	4	4
$ISE_{1}$	0.019	0.0226
$ISE_{2}$	0.014	0.0203
$max(er_{1})$	0.0335	0.0375
$max(er_{2})$	0.0324	0.0432

Problem 2:

ISE<0.04

max(er)<0.06

**Figure 12.**
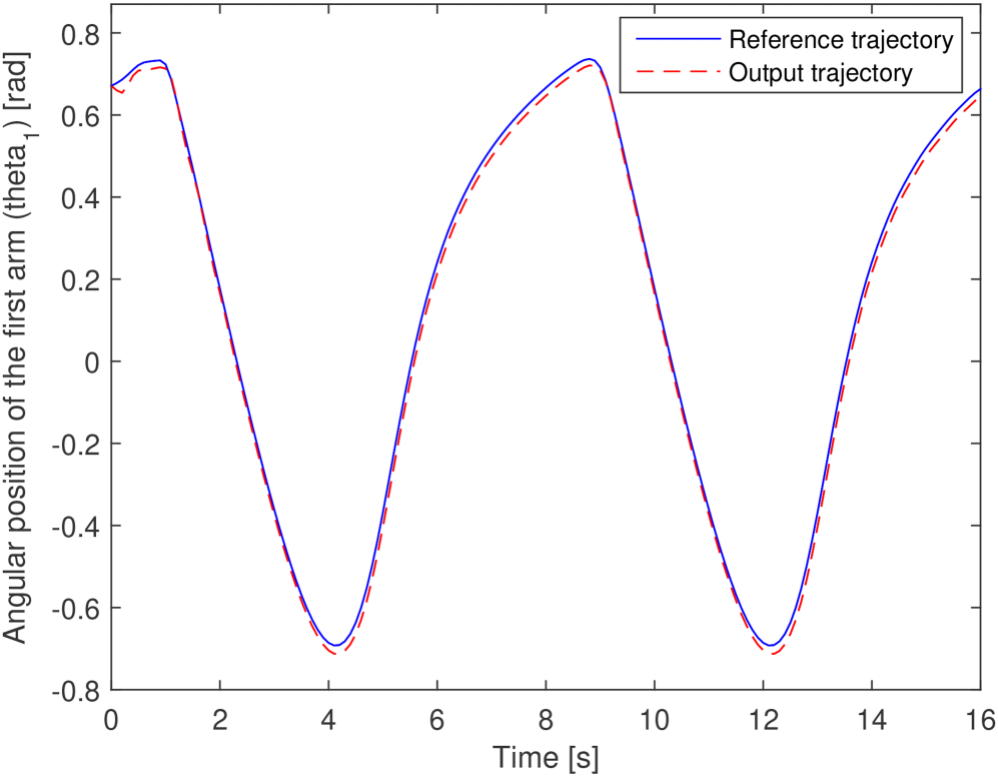
Trajectory tracking of the first arm (first motor angular position).

**Figure 13.**
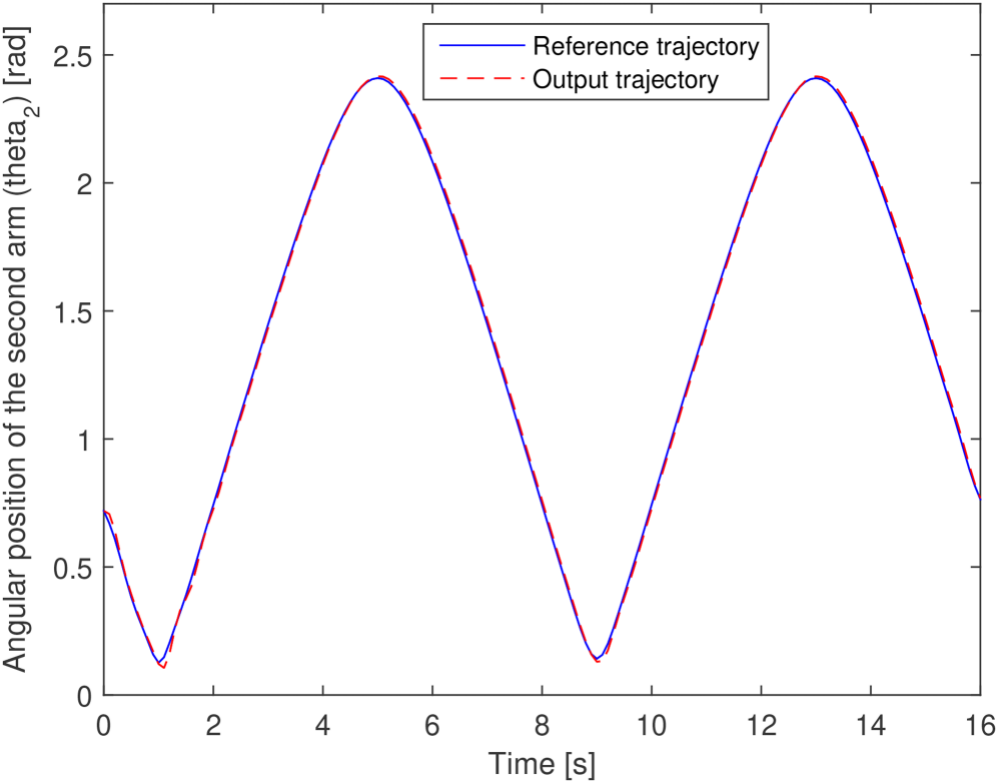
Trajectory tracking of the second arm (second motor angular position).

**Figure 14.**
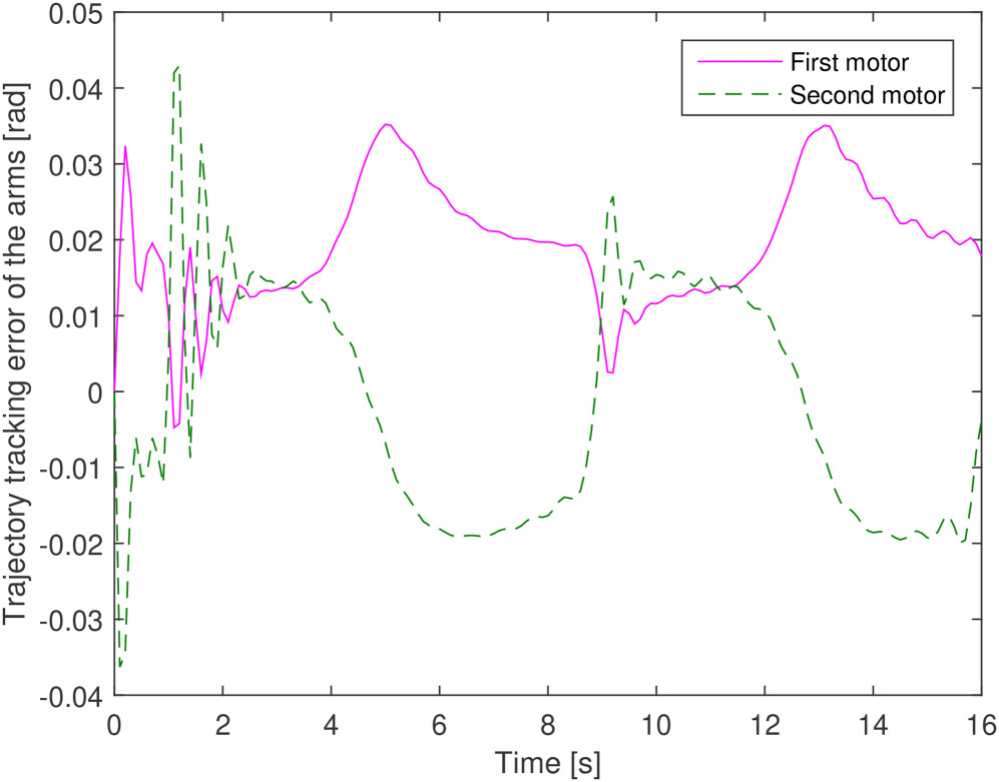
Error of trajectory tracking by the first and second manipulators.

**Figure 15.**
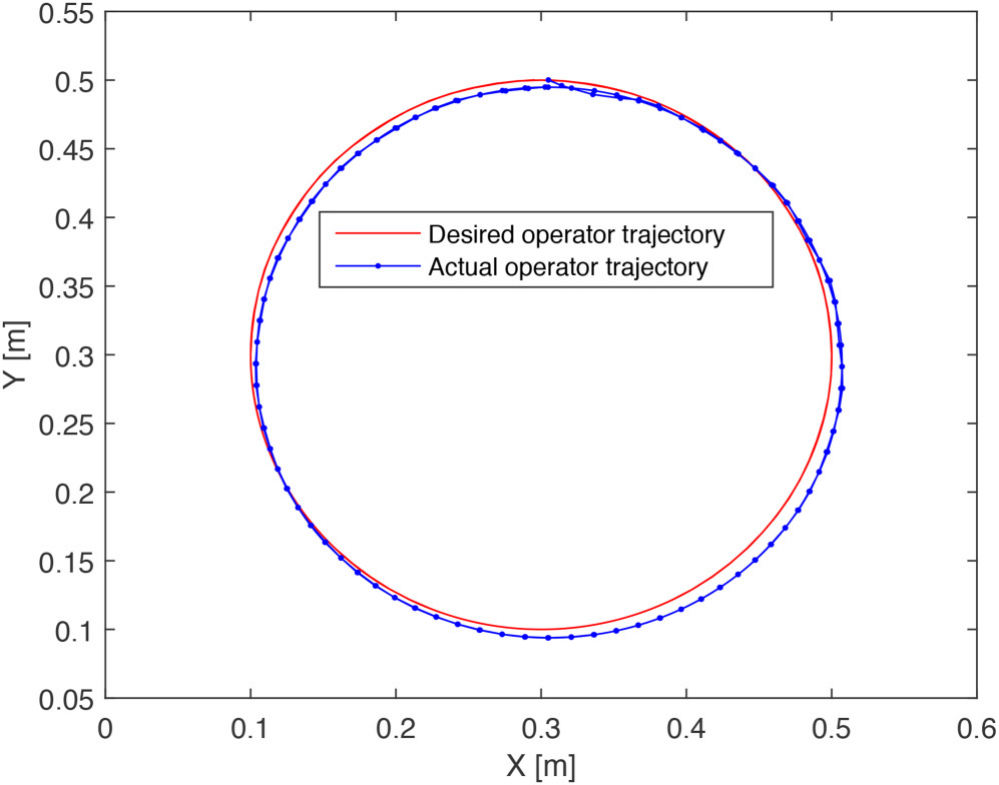
Trajectory tracking by the end effector.

The output profile shown in Figures 4 and 5 are the requirements of the system. Using the inverse kinematic approach in the static load transfer of component models, the reference position signals ( $\theta_{1}$ , $\theta_{2}$ ) for the first and second arms that are defined to be the outputs to be controlled and are derived in (22) and (23) are shown in Figures 12 and 13. The trajectory tracking performance by each arm or each actuator is also depicted in Figures 12 and 13. Figure 14 shows the error of the trajectory tracking by the 2-DOF arm. Figure 15 depicts the end effector trajectory tracking of the 2-DOF arm resulted from controlling the angles, which satisfy the control constraint as shown in Table 6.

These figures are derived from the optimal design of the system in problem 1 and show the accurate performance of the optimal PID control for the tracking of the angles of the arms and a well-performed reference position tracking of the end effector using the controlled angles. The computational time for a population size of 25 and the generation of 40 using GA is 8864.58 s which is a reasonable time for an integrated design optimization and control of mechatronic systems with 10 optimization variables; including four physical design variables and six control design variables.

In problems 1 and 2 as shown in Table 6, the entire system is optimized with GA for the objective of minimizing the volume with respect to some physical constraints (explained in Definition 8 illustrative example and in the ‘Dynamic behavioural model’ section) as well as control constraints ( ISE and max(er) ) explained in Definition 17 illustrative example. The only difference between these two problems is the control constraint boundaries ( b ).

The results in Table 6 show the large effect of the control constraints on the physical design. In problem 2, the control constraints’ boundaries are larger than the ones in problem 1, which leads to a larger physical design space solution and at the end the optimal physical design values and objective function (volume) are better in problem 2 than problem 1.

Problem 1: $v=0.0082\;m^{3}$

Problem 2: $v=0.0076\;m^{3}$

ElKhateeb and Badr^30^ studied the same case study without any physical design and dimensioning and only six control parameters are defined as optimization variables and the objective of the Bee colony algorithm optimization is defined to be mean absolute error (MAE). For comparison purposes, we select the same control parameters to range as presented by ElKhateeb and Badr^30^ and we define the control constraint to be MAE<0.04636 . MAE is given in (43):

(43) $$\begin{array}{c} \mathrm{MAE}=\frac{1}{T}\int_{0}^{T}|\left(\theta_{d1}(t)-\theta_{1}(t)\right)|+|\left(\theta_{d2}(t)-\theta_{2}(t)\right)|dt \end{array}$$

ElKhateeb and Badr^30^ assume a 2-DOF arm with specifications of $M_{1}=1\;\mathrm{kg}$ , $M_{2}=1\;\mathrm{kg}$ for the masses of the 2-DOF arm, and $l_{1}=1\;m$ , $l_{2}=1\;m$ for the lengths of the two arms. In our approach, the lengths of the arms and the motors are defined as design variables for the optimizer. Hence, there are 10 optimization parameters (four for the physical design and dimensioning and six for the control design) and we optimize the entire system for the objective of minimizing the volume. The results of the comparison are given in Table 7, which clearly shows the advantage of integrated design and control optimization in the IDIOM method that is complete in a sense that it covers both control and physical design optimization and results in a design, which is better in the final objective (volume) and the control constraint of MAE.

**Table 7.** Comparison results.

Optimization	Presented	Method by
parameters	method	ElKhateeb and Badr^30^
$l_{m1}$	0.0121m	–
$l_{m2}$	0.0894m	–
$l_{1}$	0.328m	1m
$l_{2}$	0.349m	1m
$k_{P1}$	178	200
$k_{I1}$	29	50
$k_{D1}$	3	6.9577
$k_{P2}$	188	189.496
$k_{I2}$	12	47.2071
$k_{D2}$	7	7.2797
v	$0.0083\;m^{3}$	No information provided
MAE	0.0427	0.04636

## Conclusion

Formal definitions of the proposed algorithm in the IDIOM (Integrated Design and Optimization of Mechatronic Systems) framework are presented in this paper to assist the conception of the rigorous and unambiguous definitions of the framework. The modelling capability of the IDIOM framework is improved by adding a highly non-linear complex mechatronic component as a 2-DOF arm and a new type of control component, namely an optimal PID controller. A case study is implemented and tested using the Lagrange dynamic equations for the 2- DOF arm system. The optimal PID control is implemented in the supported software framework to control each arm separately and get trajectory tracking results in the end effector of the 2-DOF arm. The system is optimized for volume and the results are compared to the achieved results of the optimal PID control using a Bee colony method which shows preciseness and satisfaction of trajectory tracking of the arms and end effector in our method. The paper covers a few technological fields such as modelling, optimization, physical design and control. The optimization is advanced to solve multidisciplinary problems where engineering features of systems from different domains are considered. The method allows simultaneous integration of mechanical, electrical and control domains. For the multidisciplinary design, different constraints with respect to the objectives and involved domains are added to the method.

The method is complete in a sense of covering physical dimension, dynamic evaluation and static properties of the system and it proves competitiveness of the control constraints results although including the physical design and constraints of the system impose solution limitations. Regarding the scalability of the proposed method and for the method to be as holistic as possible, all design parameters are required to be free variables, which would be unrealistic even for a small number of components due to the course of dimensionality.^31^ This is also true for a large number of components. A good future step would be to include design philosophies such as cooperation, agile, information management, data exchange and networking.^32^ To integrate these iterative methods with the presented method in this paper, the optimization has to be run in each iteration separately or another solution would be to implement a two-loop optimization for detailed and holistic design, respectively. However, this has to be reanalysed in detail to realize the best possible solution. To deal with incomplete information in the early stages of design, in the detailed design method in this paper static transformation models are used to derive an initial system model. A similar model/method can be considered to define initial input parameters and attributes in the holistic design loop. Even though the IDIOM framework facilitates an early-phase co-design optimization of mechatronic systems, another good future step would be to apply the presented method on a designed prototype and examine the feasibility of solution in the real world with real limitations. One another extension to the method would be to include embedded control implementation aspects in the design.^33^
